# Tracking coverage, dropout and multidimensional equity gaps in immunisation systems in West Africa, 2000–2017

**DOI:** 10.1136/bmjgh-2019-001713

**Published:** 2019-09-06

**Authors:** Oghenebrume Wariri, Bassey Edem, Esin Nkereuwem, Oluwatosin O Nkereuwem, Gregory Umeh, Ed Clark, Olubukola T Idoko, Terna Nomhwange, Beate Kampmann

**Affiliations:** 1 Vaccines and Immunity Theme, MRC Unit the Gambia at the London School of Hygiene and Tropical Medicine, Fajara, Gambia; 2 World Health Organization Country Office for Nigeria, Abuja, Nigeria; 3 Centre for International Health, Medical Centre, University of Munich, Munchen, Germany; 4 The Vaccine Centre, London School of Hygiene and Tropical Medicine, London, UK

**Keywords:** Immunisation, coverage, dropouts, inequality, West Africa

## Abstract

**Background:**

Several West African countries are unlikely to achieve the recommended Global Vaccine Action Plan (GVAP) immunisation coverage and dropout targets in a landscape beset with entrenched intra-country equity gaps in immunisation. Our aim was to assess and compare the immunisation coverage, dropout and equity gaps across 15 West African countries between 2000 and 2017.

**Methods:**

We compared Bacille Calmette Guerin (BCG) and the third dose of diphtheria–tetanus–pertussis (DTP3) containing vaccine coverage between 2000 and 2017 using the WHO and Unicef Estimates of National Immunisation Coverage for 15 West African countries. Estimated subregional median and weighted average coverages, and dropout (DTP1–DTP3) were tracked against the GVAP targets of ≥90% coverage (BCG and DTP3), and ≤10% dropouts. Equity gaps in immunisation were assessed using the latest disaggregated national health survey immunisation data.

**Results:**

The weighted average subregional BCG coverage was 60.7% in 2000, peaked at 83.2% in 2009 and was 65.7% in 2017. The weighted average DTP3 coverage was 42.3% in 2000, peaked at 70.3% in 2009 and was 61.5% in 2017. As of 2017, 46.7% of countries (7/15) had met the GVAP targets on DTP3 coverage. Average weighted subregional immunisation dropouts consistently reduced from 16.4% in 2000 to 7.4% in 2017, meeting the GVAP target in 2008. In most countries, inequalities in BCG, and DTP3 coverage and dropouts were mainly related to equity gaps of more than 20% points between the wealthiest and the poorest, high coverage regions and low coverage regions, and between children of mothers with at least secondary education and those with no formal education. A child’s sex and place of residence (urban or rural) minimally determined equity gaps.

**Conclusions:**

The West African subregion made progress between 2000 and 2017 in ensuring that its children utilised immunisation services, however, wide equity gaps persist.

**Key questions**

**What is already known?**

- Vaccination is the most cost-effective public health intervention against infectious diseases.
- Several sub-Saharan African countries continue to lag significantly behind recommended global vaccine coverage and equity targets despite having the highest burdens of vaccine-preventable diseases.

**What are the new findings?**

- Between 2000 and 2009, West African countries made significant strides at increasing national immunisation coverages and retaining children who commenced vaccination in the immunisation system.
- Coverages have plateaued in most countries since 2009 after an initial rapid increase.
- As of 2017, Burkina Faso, Cabo Verde, The Gambia, Ghana, Senegal, Sierra Leone and Togo out of the 15 West African countries had met the Global Vaccine Action Plan (GVAP) targets of third dose of diphtheria–tetanus–pertussis coverage of 90% or more.
- Guinea, Mali and Nigeria had the widest equity gaps in immunisation in West Africa, with inequalities in coverages and dropouts mostly related to poverty, low maternal education and living in certain disadvantaged regions within a country.

**What do the new findings imply?**

- Countries with low vaccination coverages and wide equity gaps in West Africa could strengthen their immunisation systems by adopting useful lessons and local solutions from their peers in the subregion who have achieved and sustained the GVAP targets on coverages and equity.
- Continued government commitment and investment in the subregion will be pivotal in reaching the children who are ‘left behind’, reducing equity gaps, and ensuring that hard-won gains are maintained especially with some Gavi-eligible countries scheduled for graduation from Gavi support in the coming years.

## Background

Vaccination is the most cost-effective intervention for the control of infectious diseases.^1^ Not only has vaccination reduced the burden and severity of vaccine preventable diseases (VPDs) such as measles and tuberculosis, but its use resulted in the eradication of smallpox in 1978.^2^ Similarly, poliomyelitis is on the verge of eradication, with only 29 reported cases of wild poliovirus across two countries globally in 2018 compared with an estimated 350 000 cases in 125 endemic countries in 1988.^3 4^ Despite this progress and the introduction of an increasing number of vaccines to the Expanded Programme on Immunisation, many children continue to go unvaccinated by their first birthday or never complete the recommended schedule.^5^ For example, in 2017, an estimated 19.9 million children globally did not receive the third dose of diphtheria–tetanus–pertussis-containing vaccine (including DTP3 and Penta3, but henceforth DTP3).^6^

Vaccination has equally been demonstrated to be a cost-effective intervention for disease control. For example, it is estimated that every dollar spent on immunisation yields more than 16 times return on investment (ROI) and when other broader benefits of vaccination are considered, the ROI rises to 48 times the cost of vaccination.^7^ Recognising its role in global health security and the cost-effectiveness of vaccination, in 2010, the WHO launched the Global Vaccine Action Plan (GVAP) aimed at ensuring that all countries worldwide achieve a DTP3 coverage of 90% by 2020 among other targets.^8^ The 2018 GVAP assessment report did, however, reveal that despite a global trend of increasing immunisation coverages rates, the achievement of these immunisation coverage indicators lagged behind, especially in low-income and middle-income countries of sub-Saharan Africa and South East Asia.^1^

In sub-Saharan Africa, particularly in West Africa, immunisation system performance and vaccination coverage rates are lower than in other regions of the world despite having the highest burden of VPDs.^9^ Additionally, entrenched intra-country equity gaps in immunisation systems, a situation characterised by marked differences in access and utilisation of immunisation services across several social and economic determinants of health, exist in West Africa.^9^ In this landscape, a subgroup of the most vulnerable, and disadvantaged population in whom an increase in vaccination uptake is necessary to ensure optimal reduction in VPD burdens will continue to be left behind. If these trends continue, hard-won gains in reducing VPD-related under-5 mortality may be halted and reversed, with a significant risk of resurgence of previously controlled infectious diseases. Understanding existing inequality gaps will be pivotal to designing effective strategies to address the needs of disadvantaged subgroups and track progress made towards achieving global targets.

In a landscape of high VPD burden and a lagging trajectory in achieving the global immunisation targets, Gavi, the Vaccine Alliance is the primary external source of assistance for immunisation in most countries of the West Africa subregion, supporting the introduction of new vaccines, and strengthening immunisation systems to improve the performance.^10^ By unpacking the immunisation system performance through the lens of coverage and equity and tracing the trajectory prior to and after the GVAP targets were introduced, we wish to highlight the key areas across and within West Africa that would benefit from governments’ and development partners’ attention. In this article, we assessed and compared the immunisation coverage, dropout and equity gaps across 15 West African countries between 2000 and 2017 in relation to the GVAP targets.

## Methods

### Study setting and context

**Figure 1:**
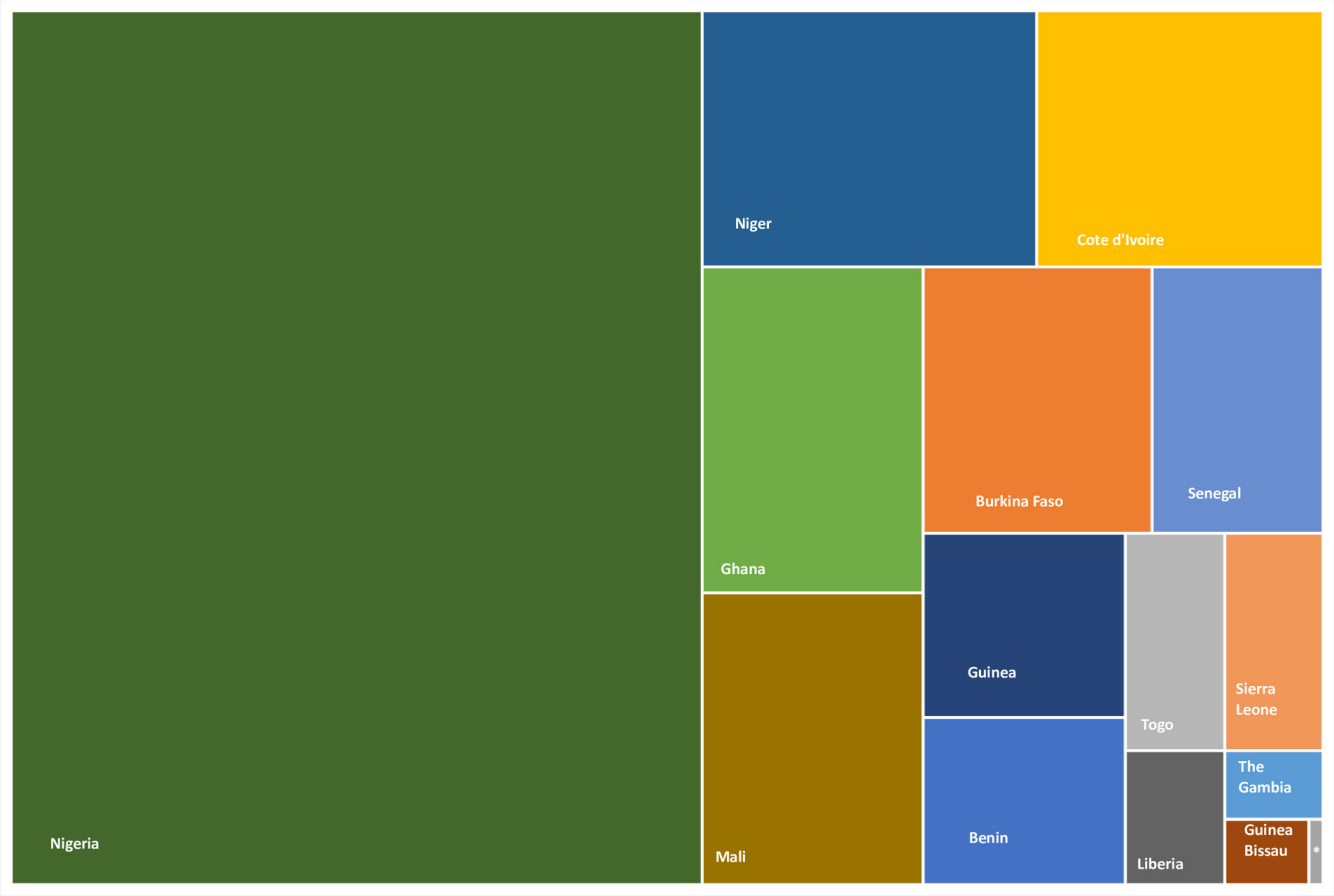
TreeMap showing the distribution of the 13,111,000 estimated surviving infants’ population across West Africa in 2017. (Source: Developed by authors based on UN population estimates of surviving infants for 2017). *Cabo Verde.

This paper set out to analyse the immunisation system performance between 2000 and 2017 across 15 West African countries whose combined estimated population in 2017 was about 372 million.^11^ In 2017, the countries had an estimated total birth cohort and surviving infant population of 13 921 000 and 13 111 000, respectively (figure 1).^12^

The West Africa subregion has a regional alliance, the Economic Community of West African States (ECOWAS) aimed at promoting integration in all fields of activity, including the health of the constituting countries. The West African Health Organisation (WAHO) is a specialised institution of ECOWAS responsible for health-related issues in the subregion. The ECOWAS is made up of 15-member countries including Benin, Burkina Faso, Cabo Verde, Cote d’ Ivoire, The Gambia, Ghana, Guinea, Guinea Bissau, Liberia, Mali, Niger, Nigeria, Senegal, Sierra Leone and Togo who have cultural and geopolitical ties, and shared economic interest.^11^ Ten of these fifteen countries were low-income countries with Gross National Income (GNI) per capita of $995 or less, whereas Cabo Verde, Cote d’Ivoire, Ghana, Nigeria and Senegal were lower-middle-income countries with GNI per capita of between $996 and 3895 based on the 2017 World Bank fiscal year classification.^13^

### Study design

We examined publicly available secondary data on Bacille Calmette Guerin (BCG), and DTP3 immunisation coverage, DTP1–DTP3 dropout rates and intra-country equity gaps, between 2010 and 2017 across 15 ECOWAS West African countries.

### Patient and public involvement

This was an analysis of publicly available secondary data and did not involve primary patients or study participants in the development of the study question, study design or conduct of the study.

### Ethical approval

As this study was based purely on secondary analysis of data and involved no primary data collection involving patients or vulnerable groups, ethical approval was neither required nor sought. The ethical procedures for the secondary data used were the responsibilities of the institutions that commissioned, funded or managed the surveys utilised. The protocol for Demographic Health Survey (DHS) was reviewed and approved by ICF Institutional Review Board, and each host country’s ethics board prior to the commencement of the surveys.^14^ The Multiple Indicator Cluster Survey (MICS) survey protocols were reviewed by country-level ethics review boards and approved according to the laws in each host country.^15^ Additionally, DHS and MICS reports that survey respondents gave written informed consent before participation.

### Measuring immunisation coverage, dropout, and equity

Immunisation systems performance is measured via multiple dimensions, including immunisation coverage, immunisation dropout, equity of coverage, the completeness of vaccines in the national schedule compared with recommended vaccines and other administrative indicators.^16^ In this paper, we measured immunisation coverage using BCG immunisation coverage and DTP3 immunisation coverage. Immunisation dropout was assessed by measuring the DTP1 to DTP3 dropout rates.

To evaluate levels of inequality in immunisation coverage, we stratified the immunisation coverage by socioeconomic and geographic determinants of inequality. These include (i) child’s sex; (ii) location of residence; (iii) maternal education; (iv) regions within a country and (v) wealth quintile according to the framework of the WHO Commission on Social Determinants of Health.^17^ Although the framework considers other dimensions such as ethnicity, migration status and other intermediate determinants, we were not able to include these indicators in the analyses because they are not routinely captured in national survey data.

### Data sources

National BCG and DTP3 vaccination coverage estimates were obtained from country-specific WHO and Unicef Estimates of National Immunisation Coverage (WUENIC) data for all 15 countries.^18^ The data extracted covered a period beginning from one-decade pre-GVAP (2000) up until the most current country-specific data as of December 2018. National and regional estimates of birth cohort and the number of surviving infants were computed using the UN Population Division population estimates, 2017 revision, which are comparable to the estimates available in WUENIC databases.^12^

To determine equity gaps in vaccination coverage, we searched the publicly available UNICEF-supported MICS and the USAID-supported DHS databases for each country’s most recent final report of either household surveys as of December 2018.^19 20^ The MICS and DHS are nationally representative, large-scale and standardised household surveys, whose methodologies allow for direct comparisons of their data. They collect and report disaggregated health data, including immunisation coverages by several socioeconomic or geographical determinants of inequality including wealth quintile, maternal education, location of residence, regions within a country and child’s sex.^21^ The detailed methodologies of MICS and DHS are described elsewhere.^21^

### Data analysis

The data generated after abstraction from the various data sources were entered into and analysed descriptively using Microsoft Excel (2016).

#### Estimating BCG immunisation coverage

BCG immunisation coverage was estimated as the percentage of children 12–23 months in each country who had received BCG.^16^ We calculated the median, and the average subregional coverage weighted by each country’s corresponding annual birth cohort population from 2000 to 2017. BCG coverage was selected as an indicator as it is considered a key determinant of access to health services.^22^

Furthermore, we illustrated the highest and lowest BCG immunisation coverage in the subregion for the corresponding year by creating two trend lines representing the country with the highest and lowest BCG coverage from 2000 to 2017. To track progress in BCG immunisation coverage, the GVAP recommended minimum coverage target of 90% was used as the reference standard.^8^

#### Estimating DTP3 immunisation coverage

The DTP3 immunisation coverage indicator was defined as the percentage of children aged 12–23 months who had received DTP3.^16^ Similar to estimating BCG immunisation coverage, we estimated the subregional DTP3 coverage trends from 2000 to 2017 by calculating and reporting the median, and average coverage weighted by each country’s corresponding annual surviving infant population. The countries with highest and lowest coverages for each corresponding year were illustrated using different trend lines. To track progress in DTP3 immunisation coverage, the GVAP recommended minimum DTP3 coverage target of 90% was used as the reference standard.^8^

#### Estimating immunisation dropout

We determined immunisation dropout rate for each country by calculating the proportion of children who dropped out from the immunisation system between DTP1 and DTP3,^22^ estimated by calculating the percentage coverage of DTP1 minus DTP3 for each country per year from WUENIC data.^18^ Similarly, we reported trends per country from 2000 to 2017, and illustrated the median and weighted average subregional trend in immunisation dropout, and the corresponding highest and lowest vaccine dropout representing the country with the highest and lowest dropout rates. To track progress in immunisation dropout, the GVAP recommended maximum dropout rate of 10% was used as the reference standard.^8^

#### Estimating unvaccinated children

Because the analysis considered West Africa as a single unit, we further highlighted the ‘hotspot’ countries where unvaccinated children lived in the subregion. We estimated the absolute number of unvaccinated children as the proportion of the 2017 country-specific number of surviving infants (number of children eligible for DTP3 vaccination) who did not receive DTP3 in 2017. We illustrated and compared this in relation to each country’s overall DTP3 coverage for the same year using a bubble map.

#### Estimating inequality in immunisation coverage and dropout

We estimated the most recent situation of intra-country inequality in BCG and DTP3 coverage, and immunisation dropout using a single summary measure of difference (between the extremes within each dimension of inequality) as a proxy for absolute inequality.^23^ This was calculated by subtracting the coverage in one subgroup from another defined a priori across the five dimensions of inequality adopted from the WHO framework.^17 23^

Several equity analyses have shown that boys, urban dwellers, children of mothers with secondary or higher education and those in the richest quintile mostly have better access to health services compared with their peers in the other extreme.^17 23^ Thus, for child’s sex, we subtracted immunisation coverage for girls from that of boys; for place of residence, urban minus rural; for maternal education, senior secondary or higher education minus pre-primary or none; and region in a country, the region with highest coverage minus region with lowest coverage. We used this method to estimate the intra-country equity gap for BCG and DTP3 immunisation coverages.

To estimate the intra-country equity gap in immunisation dropout, the first step was to calculate the immunisation dropout rate per subgroup in each of the dimensions; for example, we calculated the dropout in the male subgroup and female subgroup separately. Following this, we computed the difference in immunisation dropout between the subgroups similar to the scenario in BCG and DTP3 immunisation equity gaps calculation.

Lastly, we displayed and compared the equity gaps for BCG, DTP3 and dropout across all five dimensions in the subregion using an Equiplot (a plot for equity analysis) according to the WHO Health Equity Assessment Toolkit Plus.^23^ To track the equity gap across the five dimensions, the GVAP recommended maximum gap of 20% points between those in the poorest and richest wealth quintile was adopted as the reference standard.^8^

## Results

### Trends in BCG immunisation coverage in West Africa

**Figure 2:**
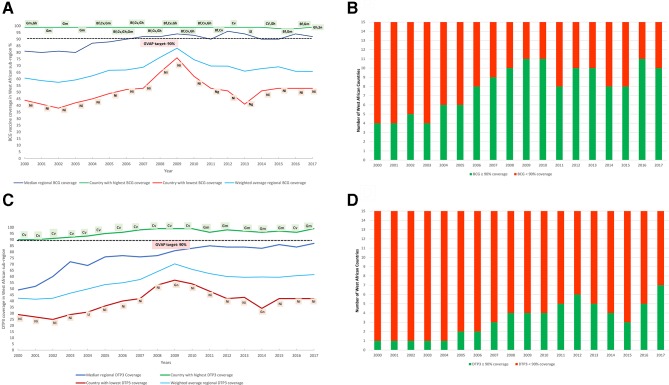
(A)Trends in sub-regional median and average weighted Bacillus Calmette-Guerin (BCG) coverage, (B) Number of countries achieving the GVAP BCG coverage target (C) Trends in sub-regional median and average weighted Diphtheria-Tetanus-Pertussis 3 (DTP3) containing vaccine coverage, (D) Number of countries achieving the GVAP DTP3 coverage target in West Africa from 2000 to 2017. Bf=Burkina Faso, Cv= Cabo Verde, Gm= The Gambia, Gn= Guinea, Gh= Ghana, Ni= Nigeria, Ng= Niger, Sn= Senegal,Sl=Sierra Leone. Data source:WHO/UNICEF Estimates of National Immunisation Coverage. All graphic representation done by authors.

There was a general trend of increasing BCG immunisation coverage across West Africa between the year 2000 and 2017. The median subregional BCG immunisation coverage increased from 81% in 2000 to 92% in 2017 (figure 2A). The weighted average subregional BCG coverage was 60.7% in 2000, peaked at 83.2% in 2009 and was 65.7% in 2017. Outlier high-performing countries include Burkina Faso, Cabo Verde, The Gambia, Ghana, Senegal and Sierra Leone who consistently recorded BCG vaccination coverage of greater than 90% since 2010 and contributed to the high median BCG immunisation coverage trend in the subregion. Nigeria, on the other hand, consistently recorded the lowest subregional BCG immunisation coverage ranging from 38% to 76% (average of 52%) between 2000 and 2017. The exception to this was in 2011 and 2013 when Niger republic had the lowest BCG vaccination coverage in the West Africa subregion (online supplementary table 1).

10.1136/bmjgh-2019-001713.supp1 Supplementary data

### Trends in DTP3 immunisation coverage in West Africa

The median subregional DTP3 coverage increased from 49% in the year 2000 to 87% in 2017. The subregion achieved a median DTP3 coverage of 83% in 2010 when the GVAP targets were set. The median subregional DTP3 coverage has however, plateaued since, with minimal yearly increase until 2017 (figure 2C). The weighted average subregional DTP3 coverage was 42.3% in 2000, peaked at 70.3% in 2009 and was 61.5% in 2017.

Twenty per cent (3/15) of countries in the subregion (Burkina Faso, Cabo Verde and The Gambia) had achieved and consistently maintained the GVAP DTP3 coverage target since 2015 while four additional countries (Ghana, Senegal, Sierra Leone and Togo) joined in 2017 (figure 2D). In contrast, Guinea, Mali and Nigeria consistently had the lowest DTP3 coverage trends in the subregion (online supplementary table II).

### Unvaccinated children in West Africa

**Figure 3:**
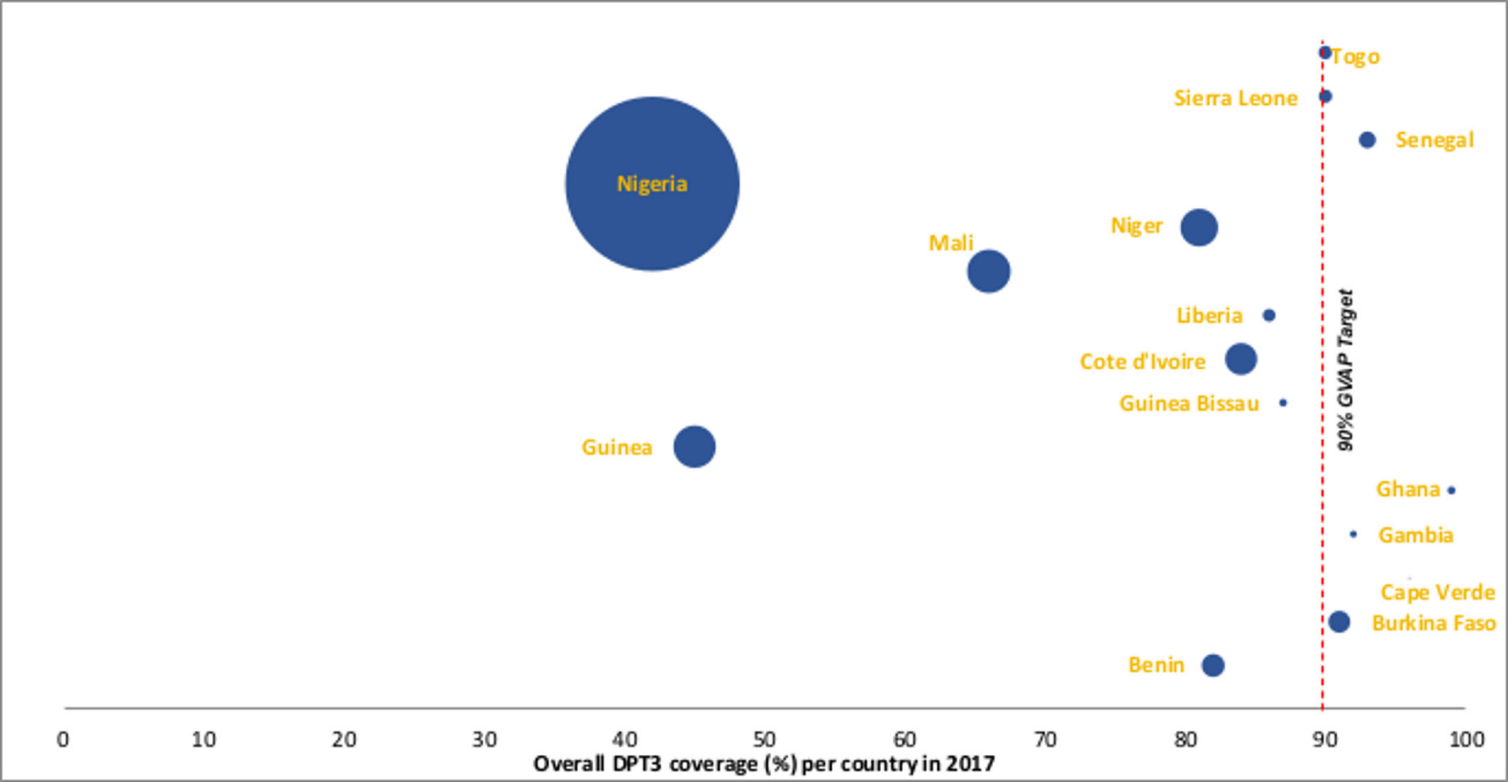
Number of children who did not receive DTP3 per country in West Africa based on the number of surviving infants in 2017 and the 2017 DTP3 coverage. The size of the bubbles is proportional to the numberof unvaccinated children in each West African country. Data source: Birth cohort data (United Nations population estimates, 2017 revision), DTP3 coverage (WUENIC). All graphic representationdone by authors.

There were an estimated 5 051 668 children who did not receive DTP3 vaccination in the 15 West African countries in 2017. Most of these children, 3 957 966 (78.3%), lived in Nigeria which accounted for 52.3% of the subregional 13 111 000 surviving infants in 2017. Countries with high DTP3 coverage typically had the lowest absolute numbers of unvaccinated children in the subregion compared with countries with low DTP3 coverage (figure 3).

### Immunisation dropout rate: trends in DTP1–DTP3 dropout across West Africa

**Figure 4:**
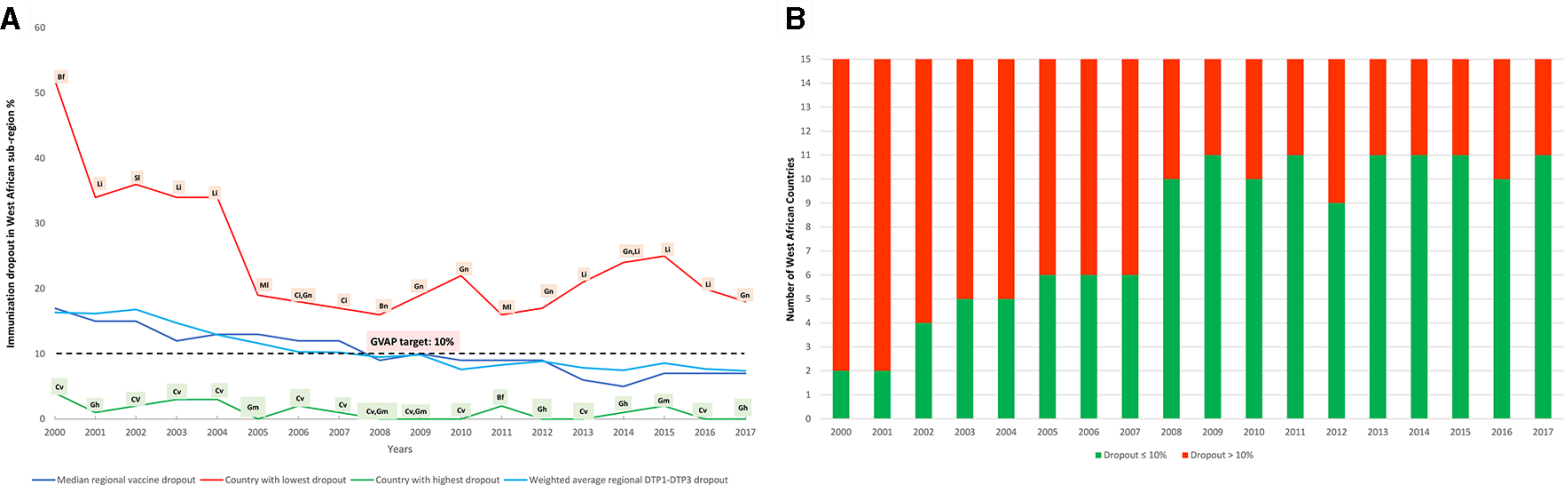
(A) Trends in dropouts from 1^st^ dose of Diphtheria-Pertussis-Tetanus-containing vaccine to 3^rd^ dose of Diphtheria-Pertussis-Tetanus-containing vaccine (DTP1-DTP3), (B) Number of countries achieving the GVAP dropout target across West Africa. Bf= Burkina Faso, Bn= Benin, Ci= Coted’Ivoire, Gh= Ghana, Gm= The Gambia, Gn= Guinea, Li= Liberia,Ml= Mali, Si= Sierra Leone. Data source: WHO/UNICEF Estimates of National Immunisation Coverage. All graphic representation done by authors.

Subregional DTP1–DTP3 dropouts reduced from a median of 16.4% in 2000 to 7.4% in 2017, meeting and maintaining the GVAP target of <10% dropout rate since 2008 (figure 4A). The weighted average subregional immunisation dropout mirrored the median subregional trends. Overall, countries with high DTP3 coverage maintained a low dropout below the recommended target. Burkina Faso, Cabo Verde and The Gambia consistently maintained dropout rate below 10% since 2005.

Four countries in the subregion (Cote d’Ivoire, Guinea, Liberia and Niger) consistently failed to meet the GVAP target of <10% dropout throughout the period under review (Figure 4B and online supplementary table 3). Nigeria which consistently had the lowest DTP3 coverage during the period under review, however, maintained a DTP1–DTP3 dropout of 10% and below since 2006.

### Equity gaps in BCG immunisation coverage

**Figure 5:**
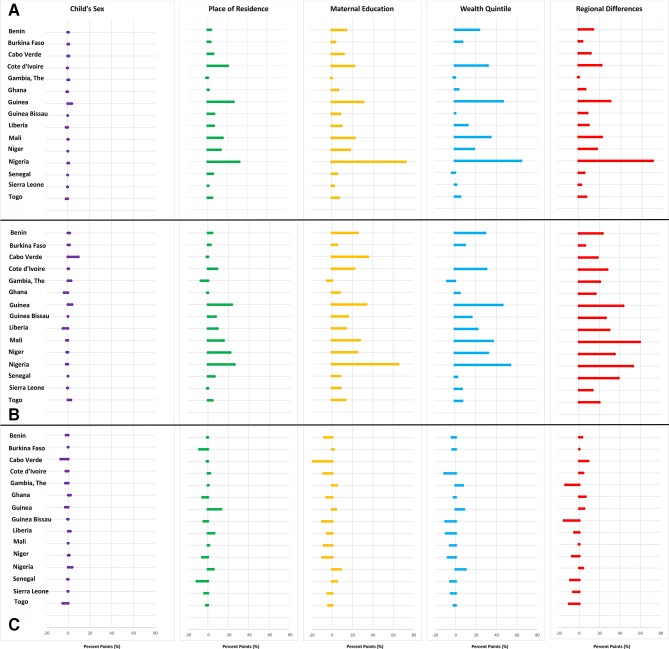
Multi-dimensional equity gaps in (A) BCG coverage, (B) DTP3 coverage and (C) DTP1-DTP3 immunisation dropout in the ‘most disadvantaged’ compared to the‘most advantaged’ children per country across West Africa. Cabo Verde’s immunisation data was not disaggregated by family wealth status. The latest available nationally representative DHS or MICS survey data used per country. Benin (MICS 2014), Burkina Faso (DHS 2010), CaboVerde (DHS 2005), Cote d’Ivoire (MICS 2016), Gambia (DHS 2013), Ghana (DHS2014), Guinea (MICS 2016), Guinea Bissau (MICS 2014), Liberia (DHS 2013), Mali(MICS 2015), Niger (DHS 2012), Nigeria (MICS 2016/17), Senegal (DHS 2017), Sierra Leone (MICS 2017), Togo (DHS 2013/14). All graphic representation done by authors.

The equity gap in BCG immunisation coverage in-country was more than the recommended 20% points or more in Cote d’Ivoire, Guinea, Mali and Nigeria across all equity dimensions except for sex (figure 5A). These same countries typically had the lowest BCG coverages across the subregion (online supplementary table 4). Contrastingly, The Gambia, Ghana and Sierra Leone with high BCG coverages of more than 95% had the narrowest equity gaps of less than 20% points across all dimensions (figure 5A, online supplementary table 4). Overall, the gender of children and location of residence (urban or rural) seemed to contribute minimally to inequalities in BCG immunisation coverage in the subregion compared with other dimensions (figure 5A).

### Equity gaps in DTP3 immunisation coverage

The equity gap in DTP3 was highest and more than 20% points in Guinea, Mali, Niger and Nigeria across all dimensions except for child’s sex (figure 5B). Again, similar to the pattern seen in the equity gaps in BCG immunisation coverage, the countries with very wide equity gap also had low DTP3 immunisation coverage compared with other countries in the subregion. Furthermore, countries with high DTP3 coverage also had narrow equity gap across all dimensions (online supplementary table 5). Overall, the inequality in DTP3 coverage in most countries was mainly related to equity gaps of more than 20% points between the wealthiest and the poorest, high coverage regions and low coverage regions, and between children of mothers with at least secondary education and those with no formal education (figure 5B).

### Equity gaps in dropout rates (DTP1–DTP3 dropout)

Overall, those living in urban areas, those in regions with the highest coverage, those in the wealthiest quintile and those with maternal secondary education or more had lower DTP1 to DTP3 dropouts compared with the other extreme. The exception was in Nigeria and Guinea where the children from the ‘most advantaged’ backgrounds had wider DTP1 to DTP3 dropout compared with the children from the ‘most disadvantaged’ backgrounds. Equity gaps in immunisation dropout rates were generally narrower compared with those in BCG and DTP coverage (figure 5C).

## Discussion

There is no doubt that significant progress has been made in improving vaccination coverage by immunisation systems across the subregion. This progress should, however, be interpreted cautiously. Although we found an increasing trend of vaccination coverage in the subregion, significant inter-country variations remain. The increase in weighted average subregional BCG and DTP3 coverage has stalled since 2009, falling below the GVAP DTP3 coverage target. For example, Burkina Faso, Cabo Verde and The Gambia, which have consistently maintained national DTP3 coverage above the GVAP recommendation since 2015, cumulatively account for less than one-tenth of the total population of surviving infants in the subregion who are eligible for DTP3 immunisation. These highly successful countries, therefore, masked the trends in the relatively poor performing countries and impacted significantly on the increasing median subregional coverage trends. This highlights the need for weighted averages at subregional levels and to avoid relying solely on medians at subregional or macro-level.

In our study, three countries, Guinea, Mali and Nigeria, accounting for >60% of the subregional surviving infant population eligible for DTP3 immunisation in 2017 persistently had the poorest and stagnant coverage trends. This is historically similar to findings in other reports from the West Africa subregion^24^ and WHO Afro region.^25 26^ These three countries are consistently ranked low on the Human Development Index (HDI), with both Mali and Guinea ranked among the 10 lowest countries in Africa on the 2016 HDI.^27^ All three countries have experienced repeated armed conflict over the past decade, in addition to specific issues such as the 2014 Ebola outbreak in Guinea, and a huge rural, nomadic and sparsely distributed terrain in Mali. Nigeria is the only country in Africa yet to be certified polio-free highlighting critical issues which impacted negatively on immunisation and routine health systems functions.^28^ These countries, therefore, harbour a large population of unvaccinated children and a cluster of the most vulnerable populations in the subregion, many of whom are continually being excluded from lifesaving interventions and are systematically missed or ‘left behind’ by immunisation systems.

Furthermore, there is a correlation between immunisation programme performance and the routine health system in which it functions. Poor immunisation system performance often highlights underlying structural weaknesses in the health system in which it operates.^29^ Issues of poorly financed health systems,^30 31^ demotivated human resource for health^32^ and poor planning for immunisation within the wider context of the health system^33^ have been reported in Mali, Guinea and Nigeria as contributory to poor coverages and dropout. However, the above factors can only partially explain the poor performance in these countries, as other relatively well-performing countries in the subregion also grapple with similar challenges.

Cabo Verde, Burkina Faso and The Gambia can be considered as ‘outlier’ highly successful countries in West Africa as they have consistently maintained DTP3 coverage above 90% since 2015 and dropout rate below 10% since 2005. The fact that these countries rank low in the HDI such as Guinea, Mali and Nigeria suggest that other context-specific issues determine the high childhood vaccination coverage and low dropout in these setting. The Gambia and Cabo Verde, for example, are relatively small countries, the majority of the population live within a convenient distance from a health facility, and the government routinely provide immunisation services via ‘trekking’ (out-reach) clinics which could partly explain the success of their immunisation system.^34^ Organisation of the health system with systematic outreach immunisation clinics has also been reported in Burkina Faso as contributory to its successful immunisation system.^35^

The subregional median and weighted average immunisation dropout rate progressively fell and maintained values well below the accepted GVAP target of at most 10% almost a decade before 2017. Overall, countries in the subregion with high temporal vaccine coverage trends generally had lower dropout rate between DTP1 and DTP3 highlighting the consistency and resilience of their immunisation systems to reach a child multiple times with the same antigen. This specific finding, thus, suggests that improving overall vaccination coverage and strengthening routine immunisation may be key in reducing vaccination dropouts.

The fact that Nigeria, with consistently poor and stagnant DTP3 coverage trends between 2000 and 2017 nevertheless achieved the GVAP target of <10% dropout between DTP1 and DTP3 since 2010 is an interesting finding, which should be viewed with caution. While the country may have been effective in delivering multiple antigens consistently, and retaining infants within its immunisation system, it may be systematically leaving behind a cluster of the most vulnerable infants who may never interface with the immunisation system. In this landscape, achieving the GVAP targets and appropriate coverage needed to provide indirect protection may be a mirage with an ever-present risk of VPDs outbreak if clear and consistent efforts are not initiated and sustained.

Beyond adequate vaccination coverages, achieving equity in national immunisation systems is a key focus of Gavi’s strategy for 2016–2020.^36^ Our study showed that Guinea, Mali and Nigeria with the lowest BCG and DTP3 coverages trends in the West African subregion also had the widest equity gaps across all measured dimensions of inequality. If countries in the subregion aim to achieve the GVAP immunisation coverage targets by 2020, this scenario of children who face multiple socioeconomic disadvantages and are potentially being left behind by immunisation systems must be halted and reversed.

The first step towards overcoming inequalities requires reliable evidence about those who are excluded in order to inform the delivery of equitable and efficient services.^37^ Similar to previous findings,^38 39^ our analysis showed that generally, the most substantial equity gaps in vaccine coverage and dropouts across West African countries were mostly related to poverty, low maternal education and living in certain regions in a country. Differential coverage between the most advantaged and the most disadvantaged subgroup in most countries was significantly more than the recommended 20% points, Gavi’s wealth-based benchmark for monitoring equity in vaccination.^40^ A child’s sex and place of residence in a rural or urban setting minimally influenced differences in vaccine coverage.

Many equity analyses including the GVAP targets use only wealth-based measure of inequality to measure gaps in vaccination uptake. We argue that this may not sufficiently reflect the complexities of social exclusion as inequalities manifest in manifold equally important dimensions as shown in this analysis. Systematic exposure to negative, multidimensional and overlapping socioeconomic determinants significantly limit the probability of families to surmount disadvantages, access preventative interventions and ultimately puts them at greater risk of poor health outcomes.^37^

Central to the targets of the GVAP, the aspirational goals of Universal Health Coverage (UHC), and the post-2015 Sustainable Development Goals (SDGs) are to ensure health and well-being for all and to reduce inequalities. Ensuring that every child born in West Africa, no matter which country they live in, or their socioeconomic background access and complete their recommended vaccinations is critical to meeting these GVAP, UHC and SDG targets. Governments, implementing and developments partners in the subregion should, therefore, design interventions targeted at the populations who are currently being left behind and reduce the barriers to immunisation to ensure that their needs are not masked behind subregional and national averages. This is even more so as DTP3 coverage though recommended by WHO is a rather conservative measure of coverage given that antigens like measles are only given post this point and remain crucial given the resurgence of these diseases globally. In addition, birth cohorts in these countries may be underestimated making the estimates of coverage overestimates.

Overall, there are important critical lessons to be learnt from the findings reported here. First, if the West Africa subregion is to improve the current realities and overcome the stalling DTP3 coverage, challenges in increasing vaccination coverage need to be addressed at subnational and national levels in a context-specific manner due to varying coverages across the subregion. Second, countries with low coverages in the subregion looking to strengthen their immunisation systems could adopt useful lessons from their peers in West Africa who have achieved the GVAP targets and have consistently maintained increasing coverage. Furthermore, continued commitment and investment in the countries with persistently high coverages will be pivotal in ensuring that the hard-won gains are maintained. This remains even more critical with some Gavi eligible countries scheduled for graduation from Gavi support in the coming years. Lastly, the stagnant subregional DTP3 coverage since 2009 highlights the point that efforts at improving coverage may yield more obvious immediate results at low coverage levels and as coverage increases, more significant high-level commitment and in-country ownership will be needed to reach the last child.^41^

There are inherent limitations to our study. First, our analysis did not consider the temporal impact of economic growth trajectory (measured by gross domestic product) and immunisation financing on the vaccine coverage trends and dropout presented here. There is no doubt that external donor funding such as from Gavi and government expenditure on immunisation, depended on economic growth, impact vaccine coverage trends. Further research on vaccine coverage in the West Africa subregion could explore this dimension which would provide a more nuanced interpretation of the evidence. Second, the inter-country equity comparison should be taken carefully because the national survey data used in our analysis were from varying years. Although the methodology for MICS and DHS are considered similar, the fact that the data compared are from the latest surveys done in different years across countries limit the extent to which direct comparison may be made. In addition, the DHS and MICS surveys rely on parental recall and vaccine card record to determine vaccination status; thus, responses could be affected by recall and ascertainment bias. While we acknowledge the limitations of the national surveys, they represent the best available population-based data covering all regions within the countries studied, thus allowing their findings to be generalisable.

Lastly, the five socioeconomic determinants used in the multidimensional equity analysis overlap in an individual and are interdependent. For example, an uneducated mother is likely to be poor and be clustered in a disadvantaged region. It may be important to state that our study’s aim was not to argue causality between the equity dimensions and vaccine uptake. Rather, it was to provide an alternative dashboard to monitor equity in immunisation in addition to differences in wealth quintile alone currently used by GVAP. We acknowledge that important equity gaps may be missed by comparing only the extremes within a dimension of equity and not among other subcategories, for example, between wealth quintiles 2 and 3. However, our approach highlights the equity gaps between the most advantaged and most disadvantaged population, and in this way, it is easy to compare the extremes of circumstances of inequality.

## Conclusions

Our study shows that the West African subregion has made significant strides between 2000 and 2017 in ensuring that its children access and use immunisation. However, despite initial rapid progress, BCG and DTP3 coverage have stalled over the last decade in most countries across the subregion, with an unlikely trajectory of achieving the GVAP targets on coverage, dropouts and equity. Furthermore, in underperforming countries, wide intra-country equity gaps in vaccine coverage and dropouts mainly related to maternal education, wealth and region of residence exist between the most advantaged and the most disadvantaged children.
