# Supplementary material for: Tracking coverage, dropout and multidimensional equity gaps in immunisation systems in West Africa, 2000–2017
Source: BMJ Glob Health. 2019 Sep 6;4(5):e001713. doi: 10.1136/bmjgh-2019-001713 (PMC6747924; doi:10.1136/bmjgh-2019-001713)
Supplement: Supplementary data [file bmjgh-2019-001713supp001.pdf]

Supplementary Table 1: Trends in Bacillus Calmette-Guerin (BCG) immunization coverage in West African countries from 2000 to 2017 in percentage.......... 2

Supplementary Table 2: Trends in Diphtheria-Pertussis-Tetanus-3 (DTP3) containing vaccine coverage in West African countries from 2000 to 2017 in percentage.......... 3

Supplementary Table 3: Vaccination utilization (DTP1-DTP3 dropout in percentage) trends in West Africa from 2000 to 2017 in percentages 4

Supplementary Table 4: BCG immunization coverage in percentages (%) by background characteristics among children 12-23 months across West African Countries (2005-2017) 5

Supplementary Table 5: DTP3-containing vaccine coverage in percentages (%) by background characteristics among children 12-23 months across West African Countries (2005-2017) 6

Supplementary Table 6: Vaccine utilization (DTP1-DTP3 dropout) in percentages by background characteristics among children 12-23 months across West African Countries (2005-2017) 7

Wariri O, *et al. BMJ Global Health* 2019; 4:e001713. doi: 10.1136/bmjgh-2019-001713

Supplementary Table 1: Trends in Bacillus Calmette-Guerin (BCG) immunization coverage in West African countries from 2000 to 2017 in percentage

Country	2000	2001	2002	2003	2004	2005	2006	2007	2008	2009	2010	2011	2012	2013	2014	2015	2016	2017
**Benin**	92	91	90	90	89	88	93	97	96	93	90	88	96	95	96	96	96	96
**Burkina Faso**	81	80	90	83	99	99	99	99	99	99	99	99	96	96	98	98	98	98
**Cabo Verde**	96	96	97	98	99	99	99	99	99	99	99	99	99	94	99	94	96	98
**Cote d'Ivoire**	80	80	79	78	71	85	77	94	91	95	91	74	93	90	84	79	95	92
**Gambia, The**	99	99	99	99	99	99	97	97	95	94	92	90	98	98	96	98	98	94
**Ghana**	99	94	92	92	92	99	99	99	99	99	99	98	98	98	99	97	94	99
**Guinea**	81	81	80	80	79	80	81	84	87	91	93	82	77	71	66	72	72	72
**Guinea Bissau**	77	79	82	84	87	89	90	92	93	94	93	92	94	94	94	94	94	94
**Liberia**	78	73	69	64	60	76	77	77	85	87	73	85	85	87	73	74	97	87
**Mali**	69	75	82	74	76	83	86	86	86	84	82	84	77	70	73	73	73	73
**Niger**	50	53	56	58	61	64	69	73	78	86	81	53	85	41	83	88	91	91
**Nigeria**	44	41	38	42	45	49	52	53	65	76	62	57	51	51	51	53	53	53
**Senegal**	89	89	70	77	95	92	94	96	98	97	97	97	97	97	95	95	97	99
**Sierra Leone**	74	63	76	89	86	85	83	82	91	96	97	97	98	99	90	90	92	90
**Togo**	84	83	81	79	91	96	96	91	92	91	97	97	97	97	79	86	79	75
**Regional Median**	79.5	80.2	80.9	81.2	83.6	86.6	86.8	88.7	90.7	91.8	89.9	86.9	90.3	86.5	86.1	86.4	88.0	87.7

Data Source: Data source: WHO/UNICEF Estimates of National Immunization Coverage.

Wariri O, *et al. BMJ Global Health* 2019; 4:e001713. doi: 10.1136/bmjgh-2019-001713

Supplementary Table 2: Trends in Diphtheria-Pertussis-Tetanus-3 (DTP3) containing vaccine coverage in West African countries from 2000 to 2017 in percentage

Country	2000	2001	2002	2003	2004	2005	2006	2007	2008	2009	2010	2011	2012	2013	2014	2015	2016	2017
**Benin**	78	76	75	73	72	70	74	82	75	79	76	75	80	77	78	82	82	82
**Burkina Faso**	45	62	69	79	79	82	86	89	93	92	91	91	90	88	91	91	91	91
**Cabo Verde**	90	90	91	92	93	95	96	98	99	99	99	90	94	93	95	93	96	96
**Cote d'Ivoire**	65	66	64	61	67	76	77	76	74	81	85	62	82	75	73	80	82	84
**Gambia, The**	80	87	87	87	87	95	95	95	96	98	97	96	98	97	96	97	95	92
**Ghana**	88	79	78	80	80	84	84	94	93	94	94	91	92	90	98	88	93	99
**Guinea**	46	50	53	57	60	59	57	63	60	57	64	63	53	44	34	45	45	45
**Guinea Bissau**	49	53	57	60	64	68	71	74	77	80	83	86	87	87	87	87	87	87
**Liberia**	46	42	39	35	31	60	60	65	75	81	70	77	80	76	50	52	79	86
**Mali**	43	49	61	63	69	77	78	74	74	73	73	66	65	64	66	66	66	66
**Niger**	34	36	39	41	43	45	51	57	67	71	70	75	71	70	75	75	80	81
**Nigeria**	29	27	25	29	33	36	40	42	53	63	54	48	42	43	43	42	42	42
**Senegal**	52	52	60	73	87	84	89	94	88	86	89	92	91	92	89	89	93	93
**Sierra Leone**	44	38	53	73	65	65	64	64	77	84	86	89	91	92	83	86	84	90
**Togo**	64	50	59	72	71	82	84	82	81	78	83	85	84	84	87	88	89	90
**Regional Median**	58	60.2	64.1	67.4	68.6	73.3	74.7	77.7	79.7	81.1	80.9	79.8	80.9	79.4	77.7	78.2	80.8	82.4

Data Source: Data source: WHO/UNICEF Estimates of National Immunization Coverage.

Supplementary Table 3: Vaccination utilization (DTP1-DTP3 dropout in percentage) trends in West Africa from 2000 to 2017

Country	2000	2001	2002	2003	2004	2005	2006	2007	2008	2009	2010	2011	2012	2013	2014	2015	2016	2017
**Benin**	11	12	12	13	13	14	15	12	16	10	11	10	9	9	8	4	4	4
**Burkina Faso**	52	31	20	11	11	9	5	3	4	1	2	2	4	6	4	4	4	4
**Cabo Verde**	4	4	4	3	3	2	2	1	0	0	0	9	4	0	4	4	0	1
**Cote d'Ivoire**	20	15	12	10	6	7	18	17	15	14	10	13	11	14	20	19	16	15
**Gambia, The**	17	10	9	10	9	0	3	3	0	0	2	3	1	2	2	2	4	1
**Ghana**	8	2	6	7	8	4	3	2	3	2	2	3	0	4	1	9	1	0
**Guinea**	15	14	15	14	15	16	18	13	4	19	22	13	17	20	24	18	18	18
**Guinea Bissau**	24	23	22	21	20	18	17	15	14	12	11	9	8	8	8	8	8	8
**Liberia**	34	34	34	34	34	15	15	15	10	9	10	9	12	21	24	25	20	13
**Mali**	18	29	26	28	25	19	13	15	11	9	8	14	10	5	7	7	7	7
**Niger**	12	12	12	12	13	12	12	11	9	10	13	11	16	18	17	14	12	12
**Nigeria**	13	15	18	16	13	12	9	10	10	10	6	8	9	6	5	7	7	7
**Senegal**	27	27	10	8	8	13	10	3	6	8	7	5	5	4	5	5	3	4
**Sierra Leone**	20	20	36	17	17	15	15	13	7	10	9	8	7	6	5	9	13	8
**Togo**	16	13	16	11	12	9	7	12	11	15	14	9	10	10	4	4	4	2
**Regional Median**	18.8	15.7	15.3	13.8	13.1	10.7	10.6	9.6	7.9	8.5	8.5	8.5	8.2	8.8	9.1	9.1	7.9	6.6

Data Source: Data source: WHO/UNICEF Estimates of National Immunization Coverage

Wariri O, *et al. BMJ Global Health* 2019; 4:e001713. doi: 10.1136/bmjgh-2019-001713

Supplementary Table 4: BCG immunization coverage in percentages (%) by background characteristics among children 12-23 months across West African Countries 2005-2017

	Sex		Residence		Maternal Education		Wealth Quintile		Regions			
**Country**	**Female**	**Male**	**Rural**	**Urban**	**None**	**Secondary+**	**Poorest**	**Richest**	**Lowest**	**Highest**	***Overall***	**Data source**
**Benin**	89.4	90.1	88.5	91.5	86.0	99.5	74.9	98.2	73.7	96.9	***89.8***	MICS 2014
**Burkina Faso**	98.0	97.0	96.0	98.5	95.9	99.1	91.6	98.5	90.0	100.0	***96.5***	DHS 2010
**Cabo Verde**	96.0	97.1	93.7	99.1	88.8	100.0	*	*	75.0	100.0	***96.5***	DHS 2005
**Cote d'Ivoire**	78.6	78.1	70.3	90.4	71.6	92.9	61.5	93.2	58.2	87.3	***78.3***	MICS 2016
**Gambia, The**	98.3	99.4	99.5	98.1	99.0	98.4	99.0	97.7	95.1	100.0	***98.9***	DHS 2013
**Ghana**	97.2	96.3	96.4	97.2	93.0	99.0	95.7	98.9	92.1	100.0	***96.8***	DHS 2014
**Guinea**	69.6	73.3	62.3	88.3	65.2	95.0	45.9	92.2	52.1	92.9	***71.5***	MICS 2016
**Guinea Bissau**	93.5	93.5	91.1	97.4	90.7	98.7	89.4	89.6	87.2	99.1	***93.5***	MICS 2014
**Liberia**	94.7	93.1	90.7	96.7	89.5	98.7	86.8	98.9	78.9	97.9	***93.9***	DHS 2013
**Mali**	72.3	72.9	69.8	84.7	68.9	90.6	56.7	91.0	41.3	93.6	***72.6***	MICS 2015
**Niger**	83.9	84.1	82.1	95.1	82.3	99.4	74.9	93.2	74.6	96.1	***84.0***	DHS 2012
**Nigeria**	53.0	54.0	43.0	75.0	23.9	93.9	23.2	87.1	30.0	85.6	***53.5***	MICS 2016/17
**Senegal**	95.4	95.2	93.4	98.4	93.5	98.6	95.8	93.0	65.8	100.0	***95.3***	DHS 2017
**Sierra Leone**	96.7	96.4	96.3	96.9	96.1	98.1	96.7	97.8	91.8	100.0	***96.5***	MICS 2017
**Togo**	96.0	94.5	93.7	97.9	92.1	98.9	93.4	98.4	90.6	98.4	***95.3***	DHS 2013/14

+The latest available nationally representative survey data used. **DHS**= Demographic and Health Survey, **MICS**= Multiple Indicator Cluster survey. *No data reported for wealth quintile

Wariri O, *et al. BMJ Global Health* 2019; 4:e001713. doi: 10.1136/bmjgh-2019-001713

Supplementary Table 5: DTP3-containing vaccine coverage in percentages (%) by background characteristics among children 12-23 months across West African Countries 2005-2017

	Sex		Residence		Maternal Education		Wealth Quintile		Regions			
**Country**	**Female**	**Male**	**Rural**	**Urban**	**None**	**Secondary+**	**Poorest**	**Richest**	**Lowest**	**Highest**	***Overall***	**Data source**
**Benin**	72.8	74.5	71.9	75.8	69.4	94.4	59.8	88.8	61.6	84.6	***73.6***	MIC 2014
**Burkina Faso**	88.8	90.3	89.1	91.5	88.8	93.6	83.4	92.9	88.0	93.7	***89.5***	DHS 2010
**Cabo Verde**	79.7	89.2	85	83.9	55.1	90.4	*	*	75.0	93.1	***84.4***	DHS 2005
**Cote d'Ivoire**	67.5	68.3	64.4	73.2	60.2	81.9	55.6	85.6	51.7	79.3	***67.9***	MICS 2016
**Gambia, The**	86.2	89.1	90.9	84.0	89.6	84.9	90.0	82.7	76.1	96.5	***87.7***	DHS 2013
**Ghana**	90.3	86.9	88.8	88.1	86.7	94.3	87.4	91.9	80.7	96.7	***88.5***	DHS 2014
**Guinea**	37.6	41.4	31.4	54.5	32.5	66.0	17.9	63.6	17.5	60.8	***39.6***	MICS 2016
**Guinea Bissau**	82.9	83.0	80.1	87.5	78.0	93.5	74.6	90.5	72.2	98.2	***82.9***	MICS 2014
**Liberia**	73.8	69.2	75.7	66.6	67.9	81.5	57.9	79.4	52.0	81.5	***71.4***	DHS 2013
**Mali**	56.2	54.9	52.6	67.7	51.9	79.2	39.6	76.0	21.0	80.5	***55.5***	MICS 2015
**Niger**	68.6	67.5	64.9	86.3	65.4	90.1	52.4	76.0	52.4	87.2	***68.1***	DHS 2012
**Nigeria**	34.1	32.6	25.3	50.8	8.9	73.8	10.2	63.3	13.7	66.4	***33.3***	MICS 2016/17
**Senegal**	91.9	92.0	89.6	95.8	89.4	97.3	86.9	88.8	60.2	98.8	***92.0***	DHS 2017
**Sierra Leone**	85.1	84.6	85.1	84.3	83.2	91.6	84.7	91.2	79.1	92.2	***84.9***	MICS 2017
**Togo**	81.4	84.2	81.2	85.5	75.4	88.4	84.0	90.9	75.3	95.2	***82.8***	DHS 2013/14

+The latest available nationally representative survey data used. **DHS**= Demographic and Health Survey, **MICS**= Multiple Indicator Cluster survey. *No data reported for wealth quintile

Supplementary Table 6: Vaccine utilization (DTP1-DTP3 dropout) in percentages by background characteristics among children 12-23 months across West African Countries 2005-2017

	Sex		Residence		Maternal Education		Wealth Quintile		Regions			
**Countries**	**Female**	**Male**	**Rural**	**Urban**	**None**	**Secondary+**	**Poorest**	**Richest**	**Lowest**	**Highest**	**Overall**	**Data source**
**Benin**	13.5	11.5	12.7	12.3	13	5.4	11.6	7.9	11.8	14.4	**12.6**	MIC 2014
**Burkina Faso**	4.9	4.7	4.9	4.7	4.8	6.4	7.3	4.3	2.4	2.5	**4.7**	DHS 2010
**Cabo Verde**	13	6.1	6.7	12.1	33.7	9.6	*	*	0	8.7	**9.6**	DHS 2005
**Cote d'Ivoire**	15.5	13.3	14	14.9	17.6	9.2	15.8	4.9	17.3	21	**7.6**	MICS 2016
**Gambia, The**	11.7	9.1	7.6	13.5	8.9	13.2	8.2	15	16.6	3.1	**10.4**	DHS 2013
**Ghana**	7	9.1	8.4	7.8	8.4	3.1	8.4	6.6	5.3	11.3	**8.1**	DHS 2014
**Guinea**	25.2	22.5	22.7	25.8	22.9	26.6	17.9	25.9	21.9	26.6	**23.8**	MICS 2016
**Guinea Bissau**	11.2	10.5	12.1	8.9	13.2	3.7	15.3	5.4	14.9	0	**10.9**	MICS 2014
**Liberia**	18.9	20.9	20.8	19.1	20.1	15.3	26.1	16.7	22.9	18.2	**19.9**	DHS 2013
**Mali**	17.1	17	17.5	14.1	16.8	9	16.5	11.1	9.8	9.4	**17.1**	MICS 2015
**Niger**	17.1	18.6	19.7	7.6	18.9	9.5	25.6	18.1	14.4	7.5	**18.1**	DHS 2012
**Nigeria**	13.6	17.2	14	18.5	10.3	18.5	9.4	19	11.6	15.2	**15.4**	MICS 2016/17
**Senegal**	4.9	4.2	6	2.2	1.1	5.7	9.5	4.3	10.5	1.8	**4.5**	DHS 2017
**Sierra Leone**	9.7	9.2	9.4	11.4	10.1	5.7	10.4	5.9	11.9	6.1	**5.9**	MICS 2017
**Togo**	12.9	7.9	9.7	11.4	13	9.3	8.6	6.7	12.9	3	**10.4**	DHS 2013/14

+The latest available nationally representative survey data used. **DHS**= Demographic and Health Survey, **MICS**= Multiple Indicator Cluster survey. *No data reported for wealth quintile

Wariri O, *et al. BMJ Global Health* 2019; 4:e001713. doi: 10.1136/bmjgh-2019-001713
